# SARS to novel coronavirus – old lessons and new lessons

**DOI:** 10.1017/S0950268820000254

**Published:** 2020-02-05

**Authors:** Brian McCloskey, David L. Heymann

**Affiliations:** 1 Centre on Global Health Security, Chatham House, Royal Institute of International Affairs, London, UK; 2 London School of Hygiene and Tropical Medicine, London, UK

**Keywords:** Emerging Infectious Diseases, epidemic Response, outbreak Response, lessons learned, SARS, coronavirus, novel

## Abstract

The response to the novel coronavirus outbreak in China suggests that many of the lessons from the 2003 SARS epidemic have been implemented and the response improved as a consequence. Nevertheless some questions remain and not all lessons have been successful. The national and international response demonstrates the complex link between public health, science and politics when an outbreak threatens to impact on global economies and reputations. The unprecedented measures implemented in China are a bold attempt to control the outbreak – we need to understand their effectiveness to balance costs and benefits for similar events in the future.

## Introduction

On 29 December 2019 clinicians in a hospital in Wuhan City, China noticed a clustering of cases of unusual pneumonia (with the first case identified at that time on 12 December) with an apparent link to a market that sells live fish, poultry and animals to the public. This event was reported to the World Health Organisation (WHO) on 31 December [1]. Within 4 weeks, by 26 January 2020, the causative organism had been identified as a novel coronavirus, the genome of the virus had been sequenced and published, reverse transcription polymerase chain reaction tests had been developed, the WHO R&D Blueprint had been activated to accelerate diagnostics, therapeutics and vaccine development and a candidate vaccine was ready for initial laboratory testing. Currently Chinese health authorities are building a 1000 bed hospital in Wuhan in 10 days.

By 26 January also, almost 50 million people in Wuhan and neighbouring cities had effectively been placed in quarantine while the WHO had determined that the event should not yet be declared as a Public Health Emergency of International Concern (PHEIC) [2] and had recommended no specific travel restrictions. The WHO have emphasised the importance of exit screening at ports in countries showing transmission of the novel coronavirus and have provided guidance for countries implementing entry screening at airports while acknowledging that evidence for the effectiveness of entry screening is equivocal.

This response is one of the swiftest, coordinated global responses to an emerging infectious disease the world has seen in modern times, but is it the appropriate response, will it be effective and is it sustainable?

## Epidemiology summary

According to the situation report published by the WHO on 28 January 2020 [3], a total of 2798 confirmed 2019-nCoV cases have been reported globally; of these, 2761 cases were from China, including Hong Kong (8 cases), Macau (5) and Taipei (4). Thirty-seven confirmed cases have been reported outside of China in eleven countries in Europe, North America, Australia and Asia; of these 37 exported cases, 36 had a travel history from China or an epidemiological link to a case from China. Of the confirmed cases in China, 461 have been reported as severely ill, with 80 deaths to date.

## Analysis

This outbreak and the response to it illustrate some key issues about how global preparedness and response capacity for outbreaks have evolved over almost two decades since the severe acute respiratory syndrome (SARS) epidemic of 2002/3 and what lessons have, or have not, been learned. It also raises questions about the impact these lessons have had on the way agencies and governments respond to these events and about the role of the WHO and the International Health Regulations (IHR).

### Coordination

One of the critical lessons from the SARS experience was the absolute necessity to be able to coordinate the international resources that are available in an outbreak and to get them focussed on identifying priorities and solving problems. The WHO established the means to do this for SARS and it has since been further developed and integrated into global preparedness, especially after the West Africa Ebola epidemic. Organisations such as the Global Outbreak Alert and Response Network (GOARN), the Coalition for Epidemic Preparedness Innovations (CEPI), the Global Research Collaboration For Infectious Disease Preparedness (GloPID-R) and the Global Initiative on Sharing All Influenza Data (GISAID) have been supported by the WHO Research Blueprint and its Global Coordinating Mechanism to provide a forum where those with the expertise and capacity to contribute to managing new threats can come together both between and during outbreaks to develop innovative solutions to emerging problems. This global coordination has been active in the novel coronavirus outbreak. WHO's response system includes three virtual groups based on those developed for SARS to collate real time information to inform real time guidelines, and a first candidate vaccine is ready for laboratory testing within 4 weeks of the virus being identified.

### Reporting

Another key factor in successfully preventing and managing emerging threats is the rapid and transparent sharing of information between countries and agencies. There was extensive criticism of China for its perceived failure to share information about the emerging SARS infection early enough in the outbreak to allow countries to prepare and respond. There were similar concerns about information sharing as Middle East Respiratory Syndrome (MERS) emerged and evolved in the Middle East in 2012, particularly in Saudi Arabia, and about the emergence of Ebola in West Africa in 2014.

On this occasion information sharing seems to have been rapid and effective (while recognising that the information available in the early stages of an outbreak is always less than the global community would like). The WHO was notified of the original clustering within days and the full genomic sequence of the new virus was published less than 2 weeks after the cluster was first detected. The WHO has expressed its satisfaction with the actions of the Chinese authorities in sharing information with the WHO.

### Journalists and risk communication

Working with journalists and the media to help them understand the science and epidemiology, particularly in a fast moving event, will improve risk communication to the public and reduce inappropriate concerns and panic.

While reporting of this outbreak shows signs of the efforts of epidemiologists, infectious disease experts, national and international public health agencies and others engaging with journalists, there are also signs that this is not yet achieving it's goal. For example, the public perception is that the increase in case numbers reported daily by the Chinese authorities represents a daily escalation in the epidemic while the reality is that these numbers are also the result of active, aggressive, case finding in China and some of these cases are ‘old’ cases newly recognised as being due to the novel coronavirus. Similarly the virus is usually described by the media as ‘deadly’ and although this is true in the sense that it has caused deaths, the nuances of uncertain case fatality rates in the early stages of an outbreak are not being communicated. The current estimated case fatality rate seems to be around 3% which is significant but not comparable to the 10% rate for SARS or 34% reported for MERS. These misperceptions are still driving public anxiety.

### Informal reporting

To supplement formal reporting mechanisms between countries and with WHO (including the IHR), the use of informal mechanisms such as media and social media reports was advocated in the light of the SARS experience. There are now globally several systems that provide collated information from informal reporting including networks of experts and scanning of media and social media. These contribute to, and amplify, epidemic intelligence and are being integrated with national and international surveillance systems.

The value, and the challenges, of this additional source of information has been evident in the current outbreak. The value comes from ensuring that early indications of cases beyond the initial outbreak city have been detected and can supplement the global risk assessment and monitoring of the evolution of the outbreak. The challenges lie in the volume and diversity of the information available and the relative lack of verification mechanisms, such that one of these systems (ProMed) has commented that it was becoming increasingly difficult to assimilate the information being supplied [4] and to make meaningful interpretations.

### Health care workers & hospital IPC

Early in the outbreak it was reported that health workers had not been infected. This was reassuring because it is health workers who many times, and inadvertently, amplify transmission. Failure to wash hands between patients, for example, can result not only in autoinfection, but also in infection of patients hospitalised for other causes when they provide care. Autoinfection is not only a risk for the health worker, but also for their families and the communities in which they live, depending on the transmissibility and means of transmission. More recently infection, and at least one death, in health workers has been confirmed. Although not unexpected this does add to the epidemiological risk.

### Superspreading events

A characteristic of the SARS outbreak was the variability of transmissibility between cases and the occurrence of ‘superspreading events’ where a case infected significantly more contacts than the average. This was also seen with MERS in the outbreak in the Republic of Korea (RoK). In this current novel coronavirus outbreak, such superspreading events have not been documented but the epidemiology is still not clear. Confirming whether or not this is happening must be an urgent task for the Chinese investigation. Modellers have suggested reproductive rates (*R*_0_) of 3.8 (95% confidence interval, 3.6–4.0) [5] and 2.6 (1.5–3.5) [6]; *R*_0_ for SARS was estimated at around 3 in the absence of control measures [7].

### Economics

The economic impact of major outbreaks can be substantial for the affected country. This was seen clearly in SARS, MERS in RoK and Ebola in West Africa. One analyst estimates that the current coronavirus outbreak's likely impact will range from a 0.8% cut to real GDP if the epidemic is controlled within 3 months, to a 1.9% cost to GDP if the epidemic lasts 9 months [8]. This may increase substantially in the light of the extended restrictions on movement, and therefore trade and commerce, within China.

## Discussion

The emergence of a significant respiratory illness linked to a novel coronavirus represents a test of the global capacity to detect and mange emerging disease threats. Its emergence in China adds an additional dimension in the light of previous experience with SARS. The timing of the outbreak immediately before the Chinese Lunar New Year with its attendant population movements adds extra risk and urgency to the response.

The rapid sharing of information in this outbreak and the speed of the coordinated response both in the country and internationally suggest that lessons have been learned from SARS that improve global capacity. The international networks and forums that now exist have facilitated the bringing together of expertise from around the world to focus research and development efforts and maximise the impact.

At this early stage in the outbreak information remains incomplete and key clinical and epidemiological questions have not yet been answered, but the deficit seems to be due more to the constraints of investigating an emerging disease than to any unwillingness to engage and share information with partners.

There are some indications of areas where further improvement is necessary. The global media response to the unfolding events has been relatively balanced and informed but the nuances of the evolving situation have not been critically examined in partnership with the media and as a result the public perception of the risk may be exaggerated – although it of course remains possible that the outbreak will develop in a way that matches up to the perceived risk. The lack of appreciation of the uncertainties in determining a meaningful case fatality rate and the significance of ascertainment bias at the beginning of an outbreak, along with the impact of aggressive case finding on case numbers, are examples of where understanding could be improved. This is always a challenging process when balancing the resources focussed on analysing the situation on the ground with resources directed at interpreting the information for journalists but in SARS, the *R*_0_ was seen to decrease in response to information reaching the public and the public then adopting risk reduction actions [6]; so accurate public risk communication is critical to success. It would be helpful to find a forum where this can be explored with the media community after the event.

The increase in access to early information from diverse sources including media and social media adds an important dimension to identifying and tracking new events globally and is a key part of the overall epidemic intelligence system. However, it is also a potential source of disinformation. When, as has been seen in this outbreak, the volume of information coming in exceeds any capacity to collate and analyse it and to attempt to cross-reference and verify separate items, there is a risk that the information fuels speculation and media and public concern. Again there is a fine balance between information that encourages appropriate risk avoidance actions and information that encourages inappropriate actions; however the public health is usually better served by more information rather than less.

The role of a declaration of a PHEIC in managing a serious outbreak has been questioned in the light of Ebola in West Africa and in the Democratic Republic of Congo [9] and has been challenged again with this outbreak. The binary nature of a PHEIC declaration (either an event is a PHEIC or it isn't – there are no intermediate options) and the specificity of the three defined criteria for a PHEIC have caused difficulty for Emergency Committees in considering whether a given event should be a PHEIC. The lack of a clear understanding of what a PHEIC declaration is meant to achieve adds to the Emergency Committee's difficulties, as does the relative paucity of clinical and epidemiological answers at this stage of the investigation. In this instance the Emergency Committee were divided in coming to a conclusion but decided on balance that the current situation, although an emergency, should not as yet be declared a PHEIC [2]. As with Ebola in the DRC, there has been criticism of the WHO for this decision but, as with Ebola, it is not immediately clear what would be different in the response if a PHEIC was declared.

The WHO is working on improving the way in which Emergency Committees develop their advice for the Director General but, as recommended by this Emergency Committee and the post-Ebola IHR Review Committee in 2015, the development of an intermediate alert alongside WHO's risk assessment process may be helpful.

A key function of a PHEIC declaration is that it is the (only) gateway to the WHO Temporary Recommendations on possible travel and trade restrictions to limit international spread of a disease. In this case several countries globally had already implemented entry screening at airports and China had begun closing down international travel from Wuhan before the Emergency Committee had finished their deliberations. While the WHO would not, and could not, interfere with the sovereign decisions of member states, the lack of influence on travel and trade decisions could prove problematic.

Alongside the speed of the response in this outbreak, we have seen dramatic changes in the scale of the response. The imposition of very extensive quarantine measures on millions of people as an attempt to break the transmission of the virus is unprecedented. We do not know whether they will be effective; indeed we do not know how we will determine if they have been effective – what end point can we measure that will provide an answer to that question? If recent suggestions that people infected with this coronavirus may be infectious while incubating or asymptomatic, and the reports that up to 5 m people left Wuhan before the travel restrictions were imposed, are confirmed, the efficacy of these control measures will be more challenged.

Given the likely impact on at least the Chinese economy and probably the global economy, it will be important to understand the role and the effectiveness of public health measures on this scale for the future.

However, the imposition of these dramatic measures does also raise a wider question: if there is an impact from these measures, what other countries would (or could) implement such measures? Would other countries accept the self-imposed economic damage that China has accepted to try and contain this outbreak? Is it reasonable to consider that national governments would close down public transport into and out of London, New York or Paris in the week before Christmas even if it were shown to be an effective control measure?

These decisions and questions cross the interface between public health, science and politics. The response to this outbreak in China was inevitably influenced by the historical reaction to the country's response to SARS and the world's suspicion of China's lack of cooperation at that time. The current response is therefore framed within a context of not wanting to be seen to be behaving in the same way with this event.

This may indicate another impact of the SARS (and MERS and Ebola) experience on the response to subsequent outbreaks – a tendency to look at worst case scenarios and respond accordingly and a fear of ‘getting it wrong’. This can deter leaders at all levels, from outbreak teams to national governments, from making judgements when all the information they would like is not available in case those judgments turn out to be wrong when the full information becomes available.

In emergency response it is generally better to over-react and then scale back if necessary rather than under-react and then act too late. Response should be on a ‘no regrets’ basis – make the best decisions possible on the basis of the best information and science available at the time but do not judge or criticise if later information suggests a different course of action. The early response must recognise what is known and what is not known and look at what of the unknowns can reasonably be estimated by reference to previous outbreaks, similar pathogens, early reporting and modelling, etc. The risk assessment and response can then be modified and refined as information on the unknowns evolves.

Key to that approach, however, is confidence that decisions will not be criticised based on information that was not available at the time. It is also important to be ready to change decisions when the available information changes – something that both scientists and politicians can find difficult.

In that context, China should not be judged for implementing what might appear to be extreme measures but China should also be prepared to discontinue the measures quickly if evidence suggests they are not the best way to solve the problem. By closing airports the international spread from Wuhan may be decreased, but success will depend on how effective the measures really are at stopping people moving out of the affected area as well as on the behaviour of the virus. As always, only time will tell – but time is scarce.
